# The fetus in the age of the genome

**DOI:** 10.1007/s00439-021-02348-2

**Published:** 2021-08-23

**Authors:** Dagmar Schmitz, Wolfram Henn

**Affiliations:** 1 Institute for History, Theory and Ethics in Medicine, RWTH Aachen University, Wendlingweg 2, 52074 Aachen, Germany; 2 Institute of Human Genetics, Saarland University, Homburg/Saar, Germany

## Abstract

Due to a number of recent achievements, the field of prenatal medicine is now on the verge of a profound transformation into prenatal *genomic* medicine. This transformation is expected to not only substantially expand the spectrum of prenatal diagnostic and screening possibilities, but finally also to advance fetal care and the prenatal management of certain fetal diseases and malformations. It will come along with new and profound challenges for the normative framework and clinical care pathways in prenatal (and reproductive) medicine. To adequately address the potential ethically challenging aspects without discarding the obvious benefits, several agents are required to engage in different debates. The permissibility of the sequencing of the whole fetal exome or genome will have to be examined from a philosophical and legal point of view, in particular with regard to conflicts with potential rights of future children. A second requirement is a societal debate on the question of priority setting and justice in relation to prenatal genomic testing. Third, a professional-ethical debate and positioning on the goal of prenatal genomic testing and a consequential re-structuring of clinical care pathways seems to be important. In all these efforts, it might be helpful to envisage the unborn rather not as a fetus, not as a separate moral subject and a second “patient”, but in its unique physical connection with the pregnant woman, and to accept the moral quandaries implicitly given in this situation.

## Introduction

The experience of pregnancy has changed radically throughout the last century. Physicians increasingly explored ways of visualizing the unborn and measuring various parameters of physiological or pathological constitution, which in turn enabled them to act preventively or therapeutically in certain situations during pregnancy (for example in birth management). This medicalization of pregnancy not only contributed to a significant decrease in neonatal and maternal mortality but, together with other developments,^1^ it also paved the way for understanding of the fetus as an unborn yet almost personal entity. In ethical and legal terms, it is seen as more or less separate from the pregnant woman (Duden 1991) and also constitutes a new and highly interesting target for medical interventions (Smajdor 2011). Efforts to learn more about the genetic status of the unborn soon followed and prenatal genetic testing grew into an important and broadly implemented subdiscipline in prenatal care. So far, these efforts mainly resulted in ever growing diagnostic possibilities, usually without accompanying therapeutic options, with this gap concomitantly generating increasing need of counseling and decision support and characterizing a pregnancy as “tentative” (Katz Rothman 1993). The main aim of prenatal genetic testing has been to enable well-informed reproductive decision-making, but it has so far been restricted to either the targeted diagnosis of few specific, individually known and typically rare heritable conditions or screening efforts directed towards a small catalogue of rather common sporadic anomalies such as Down’s syndrome or spina bifida.

Due to a number of recent achievements, the field is now on the verge of another profound transformation into prenatal *genomic* medicine (Allison 2013; Bianchi 2012; Dondorp et al. 2016; Sabbagh and Van den Veyver 2020). This transformation is expected to not only substantially expand the spectrum of prenatal diagnostic and screening possibilities, but finally also to advance fetal care and the prenatal management of certain fetal diseases and malformations. It will provide us with more options to access and treat the formerly ‘hidden’ unborn life, the fetus, as a separate patient. But it will also come along with new and profound challenges for the normative framework and clinical care pathways in prenatal (and reproductive) medicine. This ethics review aims to highlight not only the challenging aspects of the “revolution” (Dondorp et al. 2016) in prenatal medicine, but also the already available approaches and strategies to adequately deal with them in the future.

## Genomics in prenatal medicine

DNA sequencing technologies had and continue to have a major impact not only on genetic research, but also increasingly on many associated clinical care pathways (Clarke 2014). This applies particularly to prenatal medicine (Mellis et al. 2018; Schmitz 2013).

Non-invasive prenatal testing (NIPT) for prenatal aneuploidies (based on next-generation sequencing technologies) has been termed “a success story of modern genomic medicine” which in less than a decade led to a “global transformation of prenatal care” (Bianchi and Chiu 2018). The basic idea is to analyze circulating cell-free DNA (cfDNA) in the maternal blood during pregnancy to screen for fetal aneuploidy. The cfDNA is partly of maternal origin, partly of placental origin (5–20%, depending on gestational age, with a median of 10% at 10 weeks gestation) (Wang et al. 2013) and testing is usually performed at the 10th gestational week or later. Sequencing approaches are either targeted or directed towards the whole genome and the sensitivities, specificities and (at least in a high-risk population) the positive predictive values of the tests are high for the common aneuploidies. However, it is still indispensable to confirm positive results with invasive testing (Bianchi and Chiu 2018). After the first providers started offering NIPT for the trisomies 13, 18 and 21 in the USA and in China in 2011, its worldwide implementation proceeded at an unprecedented, partly market-driven pace (Bianchi and Wilkins-Haug 2014). The effects of this process will not be fully appreciable until the test is more widely reimbursed through public funding in national health care systems (as it is already beginning) (Bunnik et al. 2019; Maxwell and O'Leary 2018). A main argument in favor of implementing the option of NIPT into prenatal care also for women with low a priori risk for a fetal aneuploidy is to diminish the risk of fetal loss through skipping invasive procedures. In fact, it is already evident that the uptake of invasive procedures like amniocentesis is decreasing as a consequence of NIPT (Warsof et al. 2015). Beyond that, the indications for which the screening is offered are extended to sex-chromosomal aneuploidies and microdeletions (Bianchi and Chiu 2018). Because of the broad acceptance of the screening procedure, its easy availability and the lack of procedure-related risks, it is feared that NIPT could undermine reproductive choice (Clarke and Wallgren-Pettersson 2019) and also lead to a significant decrease in the number of live-born infants with Down syndrome (Schmitz 2019).

A second field in prenatal medicine consecutively benefiting from the development of sequencing approaches directed towards the cfDNA of placental origin in the maternal blood is non-invasive prenatal diagnosis (NIPD) for monogenic disorders. NIPD now allows for a non-invasive and risk-free, but still definitive prenatal diagnosis of some monogenic disorders (Chitty et al. 2020a; Mellis et al. 2018; Scotchman et al. 2019). Here again, the challenge is to distinguish cfDNA of maternal and of placental origin to prevent false-negative and false-positive results, which is why NIPD for monogenic disorders caused by paternally inherited or de novo mutations was one of the earlier available clinical applications. Meanwhile, indications and options have expanded (Drury et al. 2016) and also NGS panel-based approaches are being developed and implemented,—for example to test for skeletal dysplasies (Chitty et al. 2015). Clinical implementation of NIPD is proceeding much slower than in NIPT, not least due to economic reasons as the number of patients undergoing standardized NIPT is larger by magnitudes than of those suitable for NIPD. The diagnostic offer is typically limited to families at an increased risk of a monogenic disorder, but recently also screening approaches for low-risk pregnancies have been published (Zhang et al. 2019). NIPD poses special challenges to pre- and post-test counseling to safeguard autonomous decision-making in a non-invasive diagnostic or screening procedure with barely any therapeutic option available. It is feared that an increasing influence of companies on implementation processes might lead to premature diagnostic or screening offers (Chitty et al. 2020a, b).

Third, DNA sequencing technologies are now used to analyze the whole fetal exome or genome. Although it is technically possible to sequence the cfDNA in the maternal blood to gather comprehensive information about the fetal genome (Fan et al. 2012; Kitzman et al. 2012), currently only invasive approaches, using fetal DNA from chorionic villus sampling or amniotic fluid, are clinically actionable and implemented for whole-exome or -genome sequencing. While whole-exome sequencing (WES) only analyses the protein coding regions (more than 20000 genes; about 1–2% of the genome, but 85% of known variants related to disease (Ferretti et al. 2019)), whole genome sequencing (WGS) assesses the coding as well as the bona fide non-coding and unassigned regions of the genome. Fetal WGS is not integrated in clinical care pathways at the moment (Monaghan et al. 2020), but this is expected to change in the wake of falling sequencing costs and improving analytic and bioinformatic capabilities (Best et al. 2018; Mellis et al. 2018). Fetal WES is currently used not as a primary diagnostic approach but rather as an additional diagnostic tool in pregnancies where one or more structural malformations of the fetus have been detected on ultrasound, and neither karyotyping nor standard chromosomal microarray analysis (CMA) have been informative (Monaghan et al. 2020). About 2–4% of all pregnancies show fetal structural anomalies and more than half of them remain without a diagnosis after karyotyping and CMA. In such a situation, two recent large prospective studies identified an additional diagnostic yield of fetal WES of 8.5%/10% overall and 15.4%/19% in pregnancies where the fetus had more than one anomaly (Lord et al. 2019; Petrovski et al. 2019). Considerably higher diagnostic rates of 50% or more have only been described in smaller studies of 15 or fewer, most likely highly selected cases (Best et al. 2018). Trio analysis—i.e., genome sequencing of the fetus and both parents—is expected to result in a higher diagnostic yield and is therefore recommended (International Society for Prenatal et al. 2018; Monaghan et al. 2020). The importance of information about the fetal morphologic phenotype as well as the difficulties of examining it via ultrasound are frequently highlighted (Abou Tayoun and Mason-Suares 2019; Best et al. 2018; Monaghan et al. 2020). Little is known about correlations between the phenotypes noted on fetal ultrasound and the co-present genotypic variants, which will often be coincidental but may sometimes be causally related. Fetal WES is expected to expand knowledge in this field (Chitty et al. 2020a; Ferretti et al. 2019), which in turn will help to increase the diagnostic yield of fetal DNA sequencing (Abou Tayoun and Mason-Suares 2019). However, the challenges for clinical care pathways in the context of fetal WES are manifold, not only in terms of generating and evaluating sequencing data. Information and counseling issues in exome or genome sequencing are per se extremely complex, in particular as not just one individually given genetic condition has to be addressed, but rather the wide spectrum of possible outcomes, such as asymptomatic heterozygosity and susceptibility traits for late-onset diseases. Incidental and secondary findings as well as variants of unknown significance (VUS) call for strategies of reporting and intensive pre- and post-test support (Kilby 2020). In fetal sequencing (as in the postnatal sequencing of children) the patient is not able to make her/his own decisions and results may also affect one or both parents significantly. What is special in pregnancy is the very limited timeframe available for testing, counseling and decision-making. Turnaround time, thus, is a major issue in discussing possible clinical care pathways for fetal exome/genome sequencing (Abou Tayoun and Mason-Suares 2019; Ferretti et al. 2019; Mellis et al. 2018).

## Future development

### More for less

It is widely believed that future developments in prenatal genomic testing will enhance the power of clinical diagnostics, and therefore, clinical management by combining fetal WES/WGS with non-invasive or minimally invasive sampling of fetal DNA or by offering expanded NGS panels for NIPD (Abou Tayoun and Mason-Suares 2019; Best et al. 2018; Ferretti et al. 2019; Mellis et al. 2018). The ethically delicate question of which parameters should be incorporated into these panels, within the spectrum from early-onset severe recessive conditions to late-onset neurodegenerative diseases, cannot be addressed in this paper. In any case, pregnant women or couples will potentially receive a significantly larger amount of information on a wider range of genetic conditions of the unborn (and of themselves) at an earlier gestational age, virtually “at no cost” in terms of procedure-related risks.

On a practical level, there is much debate on how a shared decision-making process and autonomous choice based on sound information can be established in the face of complexity and uncertainty caused by genomic information about a huge number of clinically unrelated diseases with frequently encountered gene variants of unknown clinical significance and a high risk of unexpected (incidental or secondary) findings (Burke and Clarke 2016; Gyngell et al. 2019; Horton and Lucassen 2019; Narayanan et al. 2018; Newson et al. 2016; Richardson and Ormond 2018). Several alternative ways of presenting the information and discussing the individual implications have been proposed for genomic testing, such as a tiered approach (Bunnik et al. 2013) or a clinician-led generic consent (Dondorp et al. 2012). For pregnant women and couples, genomic testing procedures bear the promise of minimizing the probability of severe genetic diseases of their future child. At the same time, however, they come along with a substantial risk of increased uncertainty if gene variants of uncertain significance are encountered (Richards et al. 2015), which may or may not affect the health of the future child and perhaps even the future parent(s). Counseling processes will have to reflect this ambiguity (Richardson and Ormond 2018). First empirical studies are indicating that many pregnant women or couples would want to have access to extended genomic testing in pregnancy (Kalynchuk et al. 2015; Quinlan-Jones et al. 2016; Sullivan et al. 2019). But experiences with prenatal chromosomal microarray testing, for example, suggest that findings of uncertain significance can also pose a high burden on future parents during pregnancy (Bernhardt et al. 2013). In cases with abnormal results, where no therapeutic or preventive action can be taken or the effects of such actions are regarded as unsure or insufficient (which is still the rule rather than the exception), uncertainty complicates reproductive choice. It might lead to a quick decision to terminate a wanted pregnancy even in the absence of a definitely proven impairment, with grave potential for later regrets. The special situation in pregnancy with a very limited timeframe for counseling and decision-making puts additional strains on all agents involved.

### The fetus as a patient

Defining the clinical utility of prenatal genetic testing has always been a complex endeavor with potentially two patients (Schmitz 2013). Because of missing therapeutic or preventive options, prenatal genetic testing was mainly directed towards enabling reproductive choice for the pregnant woman (or couple). This traditional focus is now about to change (Bianchi 2012; Dukhovny and Norton 2018). While reproductive choice remains an important aim, we are witnessing immense progress in surgical and non-surgical prenatal and perinatal therapy, directed towards the well-being of the fetus and the future child. Prenatal spina bifida repair has become a well-accepted and clearly beneficial intervention in selected cases (Moldenhauer and Flake 2019). For other conditions such as fetal cardiac defects or congenital diaphragmatic hernia, the superiority of prenatal surgery remains to be shown (Levy et al. 2018; Wenstrom and Carr 2014). While the mentioned surgical therapies address morphological conditions typically diagnosed by means of prenatal ultrasound, only a few first successful steps towards prenatal molecular, stem cell and gene therapy of certain monogenic diseases have been published. A German group for example reported on the successful prenatal treatment of X-linked hypohidrotic ectodermal dysplasia in three fetuses (Schneider et al. 2018). In-utero gene therapy has shown its potential in the treatment of Gaucher disease in knock-out mice (Massaro et al. 2018) and is believed to be promising in hematologic disorders and cystic fibrosis, for example (Almeida-Porada et al. 2019; Carlon et al. 2017). Early (definite) diagnosis of a genetic condition via prenatal exome/genome sequencing may allow for specific prenatal and perinatal management to prevent secondary consequences, for example a specific maternal diet in metabolic genetic conditions for the future child (Rafati et al. 2016), and might in the future even be the first step towards in utero gene editing (Rossidis et al. 2018). Such early diagnosis can also prepare an appropriate plan for birth management or neonatal follow-up in the case of complex syndromic conditions and help to avoid unnecessary or burdensome prenatal interventions (Laghmani et al. 2016). If for example fetal surgery is considered as an option in a certain morphological malformation, identification of the reason through fetal WES/WGS could help to identify risks for further health-related problems and better define the prognosis which might be important for pre-surgery decision-making.

### Blurring clinical pathways

If testing procedures continue to shift focus away from targeted genetic testing to panels for a wide range of conditions or even to whole-exome/whole-genome sequencing and, in addition, can be provided non-invasively, then some traditional distinctions between clinical pathways will blur in prenatal medicine. It will be harder to distinguish screening procedures without individual indication from targeted diagnostic procedures within individually burdened families (Munthe 2017). In addition, and even more importantly, it will no longer be possible to differentiate those procedures undertaken with the intent to inform reproductive decision-making from procedures related to potential preventive or therapeutic actions (Dondorp and De Wert 2018; Dondorp et al. 2016). This is highly relevant insofar as diagnostic offers in clinical medicine have to be evaluated in relation to the end, which they are pursuing. Clinical medicine is typically understood as a practice, which is directed towards a distinct end, primarily towards the well-being of a patient (Hofmann 2003). A diagnostic or screening offer in clinical medicine, therefore, can only be fully evaluated within a specific context, in relation to the goal of the programme and the related clinical care pathway as a whole. Depending on the respective end pursued, they raise different medical and ethical challenges. Prenatal testing for trisomy 21, for example, currently does not enable relevant preventive or therapeutic actions prenatally beyond the management of delivery and, thus, is expected to be accompanied by more extensive pre- and post-test counseling and broader informed consent requirements than, for example, rhesus factor screening in pregnancy, which has a clear preventive relevance. As soon as non-invasive WES/WGS is implemented, it will be possible to deliver both results by one single testing procedure in one single clinical pathway (“double-purpose screening” (Dondorp et al. 2016)).

## Corresponding ethical challenges

### Autonomy and justice

Irrespective of whether the goal of genomic testing in pregnancy is prevention/therapy or reproductive choice, the concept of maternal (parental) autonomy is highly relevant and challenging (Clarke 2014; Harris et al. 2018). There is no unanimously accepted definition of the concept of autonomy (O'Neill 2002) which increases the complexity of the situation and makes it difficult to interpret autonomy in the context of reproductive choices. The American philosopher Ronald Dworkin sees reproductive autonomy as grounded in “a belief in individual human dignity: that people have the moral right—and the moral responsibility—to confront the most fundamental questions about the meaning and the value of their own lives for themselves: answering to their own conscience and convictions” (Dworkin 1993). Understood this way, reproductive autonomy represents a strong notion of individual autonomy. It might serve as a justification for the termination of at least an early pregnancy, a phase of human life when it is widely accepted that the unborn does not have the same rights as persons after birth. Accordingly, in an unsolvable conflict between the pregnant woman’s fully established interests and the fetus’ only emerging vital interests, the pregnant woman’s choice prevails in nearly all legal systems worldwide. If prenatal testing reveals a serious disease in the unborn, this in all likelihood not only affects the current and future psychosocial well-being of the pregnant woman (or couple), but potentially restricts her future range of opportunities in a far more complex way than living with children usually does. In such circumstances, it can be argued that a woman should get the information necessary to decide whether she wants to accept these foreseeable restrictions to individual opportunities. This argument has traditionally served as the main justification for offering prenatal genetic testing or screening without related preventive or therapeutic options (Wilkinson 2015). In the light of the recent developments in prenatal genomic medicine towards “more for less”, however, it is increasingly questioned if the argument is still valid and can possibly be extended to the whole spectrum of conditions that can be diagnosed through fetal WES/WGS (Botkin et al. 2017; Chen and Wasserman 2017; Dondorp et al. 2016; Munthe 2015).

Two principal counter-arguments are discussed. While the first questions the permissibility of such testing, the second raises the issue of priority setting in extended prenatal genomic testing (Munthe 2017). A possible limitation to the permissibility lies within the anticipated autonomy rights of the (future) child or (future) person that are believed to be harmed by genomic testing procedures (Clarke 2014; Dondorp et al. 2016). The underlying theoretical argument frequently draws upon a supposed right to an open future, as originally formulated by Joel Feinberg (Feinberg 1980). Following Feinberg, the right to an open future encompasses a group of autonomy rights “that are to be *saved* for the child until he is an adult, but which can be violated “in advance”” (Feinberg 1980). It is far from clear if and how undeniable *rights* of the future child are to be respected in the context of new forms of prenatal genomic testing, or if, in a weaker sense, the *interests* of the future child would provide a more adequate and, if so, more flexible approach (Kopelman 2007). The same is true for the correlative duties of pregnant women, (future) parents or the state. Feinberg’s argument, so far, has mainly been applied to children (who have been born) and with regard to education and religion, but it has also been included in professional recommendations regarding prenatal testing for adult-onset conditions (Hercher et al. 2016). However, conflicts between the right of reproductive choice of the parents and the autonomy rights of future children are frequently recommended to be solved in favor of the parents to protect their reproductive rights (Hercher et al. 2016).

The second group of counter-arguments is based on considerations of justice and questions the assignment of health care resources to prenatal genomic testing. Following the influential account of Norman Daniels, entitlements in health care are believed to gain moral importance inasmuch as they arise from conditions or diseases that prevent patients from their normal range of opportunities in society (Daniels 2008). Health care resources should then be used with the intention to protect fair equality of opportunity. If access to prenatal screening is limited by financial barriers, this may result in an unfair discrimination of women with insufficient financial resources (Bunnik et al. 2019; Rolfes and Schmitz 2016). Others argue that, in the context of prenatal genomic testing, such a case could be made only when a serious fetal condition is expected to significantly limit the future range of opportunities of the pregnant woman (Stapleton et al. 2019). Accordingly, it has been held that prenatal screening and funding for prenatal screening should be drastically downscaled (Munthe 2015) and limited to serious congenital and childhood disorders (Dondorp et al. 2015). However, such a limitation is frequently rejected as arbitrary since, apart perhaps from untreatable early-onset lethal conditions, the severity of a disease or impairment can hardly be measured objectively. What is more, such categories may change rapidly, as exemplified in the current therapeutic progress in spinal muscular atrophy (Wadman et al. 2019). On a societal level, there are concerns that a publicly funded prenatal (genomic) screening policy for non-treatable or non-preventable conditions could re-emphasize eugenic ideas (Iltis 2016) and lead to a “brave new world of bespoke babies” (Shakespeare 2017).

### There can be only one (patient)?

The new and exciting prospects in prenatal diagnosis and treatment give rise to an understanding of the unborn and fetus as a patient with all related consequences for clinical care pathways in prenatal medicine (Casper 1998). We owe much insight into the theoretical basis of this concept to Laurence McCullough and Frank Chervenak, who in the 1980s began to elaborate on the professional duties towards a pregnant woman and the fetus as patients (Chervenak and McCullough 1996). Being a patient since the eighteenth century is frequently described as a social role for “the sick”, created by physicians becoming professionals (McCullough 2006). The concept emphasizes a commitment of physicians to their “responsibility to protect and promote the health-related and other interests of the individual under their care” (McCullough and Chervenak 2008). By identifying the fetus as a patient, it is given a dependent moral status which comes along with beneficence-based (not rights-based) obligations of health care professionals. The authors repeatedly stressed that in this understanding the fetus is neither required to be seen as separate from the pregnant woman, nor has an independent moral status and therefore does not restrict the rights of pregnant women (McCullough and Chervenak 2018). But the adequacy and applicability of the concept is still under considerable debate (Lyerly et al. 2008; Rodrigues et al. 2013; Schmitz et al. 2018), and convincing alternatives have not been elaborated so far.

An increasing focus on the fetus as a patient in any case seems to bear a substantial risk of neglecting the needs and interests of the pregnant woman in prenatal medicine. This is especially evident in fetal surgery (Smajdor 2011) and in the general research context of prenatal medicine (Sheppard 2016; Verweij 2018). Pregnant women are regarded as exceptionally vulnerable as research participants (Sheppard 2016). In fetal therapy trials, there is a heightened risk of therapeutic misconception, that is, a misunderstanding regarding the purpose of a trial, on behalf of the pregnant woman and a resulting misjudgment concerning the potential benefits and burdens of trial participation (Verweij 2018). Previous studies on fetal surgery frequently even failed to report on maternal outcomes (Sacco et al. 2019). Only very recently, the research interest in maternal risks of fetal surgery seems to increase (Goodnight et al. 2019; Sacco et al. 2019). The concept of the fetus as a patient, although probably highly intuitive for many health care professionals, potentially puts the status of the pregnant woman as a patient at risk. It is, therefore, of central importance for future professional-ethical research to define a normative framework for the obligations of health care professionals in pregnancy which encompasses interactions directed towards the unborn as well as towards the pregnant woman and is able to capture the moral intuitions of all agents involved.

### The duties of physicians

The medical profession is characterized by a high degree of autonomy in professional practice. The new challenges of prenatal genomic medicine, accordingly, are believed to require an intense professional-ethical analysis and debate about possible ends and adequate means to achieve them. It is not only a fundamental positioning towards a potential fetal patient that is necessary but, in addition, physicians are also required to consider the medium- and long-term implications and related professional responsibilities in genomic medicine in a much stronger way than in other fields of clinical medicine. This is especially evident for prenatal genomic medicine (Clarke 2014). What—if any—are the responsibilities of health care professionals towards a child, born after fetal WES/WGS in pregnancy (Horn and Parker 2018a, b) regarding its health, regarding its anticipatory autonomy rights or related interests? Where currently implemented clinical care pathways and the related normative framework are—in the face of the challenges described above—no longer sufficient, then health care professionals must act proactively as important agents in a concerted societal effort of revision, not least through scientific outreach activities towards policy stakeholders and the general public. It is, for example, argued that possible restrictions to fetal WES/WGS should be a professional responsibility rather than a governmental one (Berkman and Bayefsky 2017). Many commentators agree that further unbalanced adherence to the reproductive autonomy paradigm on behalf of health care professionals would put too much responsibility and burden on pregnant women and their partners, or even might be abused for illegitimate decisions to terminate a pregnancy on eugenic grounds or for fetal sex selection (Henn 2000; Kraft 2017; Mozersky and Sankar 2017). New clinical care pathways in this extremely complex field of prenatal genomic medicine will have to better distribute responsibilities among all agents involved.

The professional-ethical debate on new clinical care pathways in prenatal genetic testing is not advanced enough to draft concrete proposals but has at least started. The current public discourse on reimbursement of NIPT especially for trisomy 21 seems to reflect a growing unease, also among health care professionals, and a new awareness for the moral quandaries of prenatal genetic testing (Schmitz 2019). The British Medical Association, for example, recently argued for a formal governmental consultation process on the views of the public and of health care professionals on NIPT and an ethical review of the current practice and its potential expanded use as fetal WES/WGS (Iacobucci 2018). Special attention is paid to the long-term societal implications of the new genomic technologies in terms of justice. However, it appears equally important to discuss professional-ethical aspects of prenatal genetic testing in a narrower sense, that is, with regard to ends of professional practice (and means to achieve them). Prenatal genetic testing, for example, always came along with a substantial risk of misunderstood ends, comparable to the “therapeutic misconception” in research settings. Pregnant women tend to misunderstand prenatal genetic testing as having a therapeutic utility for the fetus (Press and Browner 1997), and not as “only” enabling reproductive choice. This problem might aggravate with further blurred clinical pathways in new prenatal genomic medicine and is to be taken into account by upcoming professional-ethical initiatives.

## Conclusion

New prenatal genomic medicine has the potential to provide future parents and doctors with much more information (of sometimes uncertain clinical significance) about the genetic status of the unborn at an earlier stage of pregnancy than ever before, and with virtually no procedure-related risks. In the future, it might allow for a whole new spectrum of prenatal preventive and therapeutic options, but for now mainly just widens the range of reproductive choice during pregnancy and increases the concomitant burdens of decision-making. To adequately address the potential ethically challenging aspects without discarding the obvious benefits, several agents are required to engage in different debates. The permissibility of fetal WES/WGS in principle will have to be examined from a philosophical and legal point of view, in particular with regard to conflicts with potential rights of future children. A second requirement is a societal debate on the question of priority setting and justice in relation to prenatal genomic testing. Third, a professional-ethical debate and positioning on the goal of prenatal genomic testing and a consequential re-structuring of clinical care pathways seems to be important. In all these efforts, it might be helpful to envisage the unborn rather not as a fetus, not as a separate moral subject and a second “patient”, but in its unique physical connection with the pregnant woman, and to accept the moral quandaries implicitly given in this situation.

## Data Availability

Not applicable.

## Footnotes

1 As an illustrative example consider the famous photo essay of the Swedish photographer Lennart Nilsson in the Life magazine in 1965 (Nilsson L. Drama of life before birth. Life. 30th April 1965).
